# Rex1/Zfp42 is dispensable for pluripotency in mouse ES cells

**DOI:** 10.1186/1471-213X-8-45

**Published:** 2008-04-24

**Authors:** Shinji Masui, Satoshi Ohtsuka, Rika Yagi, Kadue Takahashi, Minoru SH Ko, Hitoshi Niwa

**Affiliations:** 1 Laboratory for Pluripotent Cell Studies, RIKEN Center for Developmental Biology (CDB), 2-2-3 Minatojima-minamimachi, Kobe, Hyogo 650-0047, Japan; 2 CREST (Core research for Evolutional Science and Technology), Japan Science and Technology Agency, Honcho 4-1-8, Kawaguchi, Saitama 332-0012, Japan; 3 Laboratory for Development and Regenerative Medicine, Kobe University Graduate School of Medicine, 7-5-1 Kusunokicho, Chuo-ku, Kobe, Hyogo 650-0017, Japan; 4 Developmental Genomics and Aging Section, Laboratory of Genetics, National Institute on Aging, National Institutes of Health, 333 Cassell Drive, Suite 3000, Baltimore, MD 21224-6820, USA; 5 Division of Molecular Biology and Cell Engineering, Department of Regenerative Medicine, Research Institute, International Medical Center of Japan, 1-21-1 Toyama, Shinjyuku-ku, Tokyo 162-8655, Japan; 6 Division for Animal Resources and Development, Life Science Research Center, Toyama University, 2630 Sugitani, Toyama 930-0194, Japan

## Abstract

**Background:**

*Rex1*/*Zfp42 *has been extensively used as a marker for the undifferentiated state of pluripotent stem cells. However, its function in pluripotent stem cells including embryonic stem (ES) cells remained unclear although its involvement in visceral endoderm differentiation in F9 embryonal carcinoma (EC) cells was reported.

**Results:**

We showed the function of *Rex1 *in mouse ES cells as well as in embryos using the conventional gene targeting strategy. Our results clearly indicated that *Rex1 *function is dispensable for both the maintenance of pluripotency in ES cells and the development of embryos. However, *Rex1*-/- ES cells showed the defect to induce a subset of the marker genes of visceral endoderm, when differentiated as embryoid body, as found in EC cells.

**Conclusion:**

*Rex1 *should be regarded just as a marker of pluripotency without functional significance like the activity of alkaline phosphatase.

## Background

Pluripotency is the differentiation ability of a cell to give rise all embryonic and adult cell types. Studies of embryonic stem (ES) cells have revealed molecular mechanisms that govern pluripotency involving in both genetic and epigenetic mechanisms [1,2]. Three transcription factors Oct3/4, Sox2 and Nanog are regarded as pivotal regulators because the loss-of-function experiments confirmed their essential functions for maintenance of pluripotency in ES cells as well as in peri-implantation development [3-7]. Moreover, the gain-of-function experiments emphasize their function associated to pluripotency. Nanog overexpression supports self-renewal of mouse ES cells in the absence of leukemia inhibitory factor (LIF) and promote imposition of pluripotency on somatic cells after cell-fusion with ES cells [8,9], whereas ectopic expression of Oct3/4 and Sox2 with additional two transcription factors Klf4 and cMyc is sufficient to induce pluripotency in embryonic and adult fibroblast cells [10]. Oct3/4 co-operates with Sox2 to activate transcription of the target genes including Oct3/4 [11], Sox2 [12] and Nanog [13]. It has been recently shown that Sox2 is essential to maintain expression of Oct3/4 in ES cells [7], suggesting that these three transcription factors form a network to maintain pluripotency.

In addition to Oct3/4, Sox2 and Nanog, other putative transcription factors expressing pluripotent stem cells in stem-cell-specific manner have been identified. Rex1 (for reduced expression-1, also known as *Zfp42*) was first identified a gene that expresses in F9 embryonal carcinoma (EC) cells and is down-regulated after retinoic acid (RA) treatment to induce differentiation [14]. This gene encodes a C2H2 zinc-finger protein that is closely similar to Yy1, an evolutionally-conserved component of polycomb-related complex 2 [15]. Its highly-specific expression in pluripotent stem cells has been confirmed in mouse and human ES cells [16,17], making it one of the most famous markers of pluripotency tested in various stem cells such as multipotent adult progenitor cells [18] and amniotic fluid cells [19]. However, its function in ES cells has not yet been characterized well although it has been reported that a targeted deletion of *Rex1 *results in loss of the ability to differentiate into visceral endoderm induced by RA in F9 EC cells [20], and that a gene silencing by RNA interference for Rex1 results in loss of capacity to self-renew in ES cells [21].

In this paper, we report our results of functional assay of Rex1 in ES cells as well as in embryos. Over-expression of Rex1 in ES cells neither induces differentiation in the presence of LIF nor maintains self-renewal in the absence of LIF. *Rex1*-/- ES cells can be established and contribute whole embryos after blastocyst injection, indicating that they possess proper pluripotency. *Rex1*-/- mice were produced by the intercross of heterozygotes, and both male and female homozygotes were normal and fertile. Our data proofed that Rex1 is dispensable for maintenance of pluripotency beyond the shadow of a doubt.

## Results

### Generation of gain- and loss-of-function mutant ES cell lines for *Rex1*

**Figure 1:**
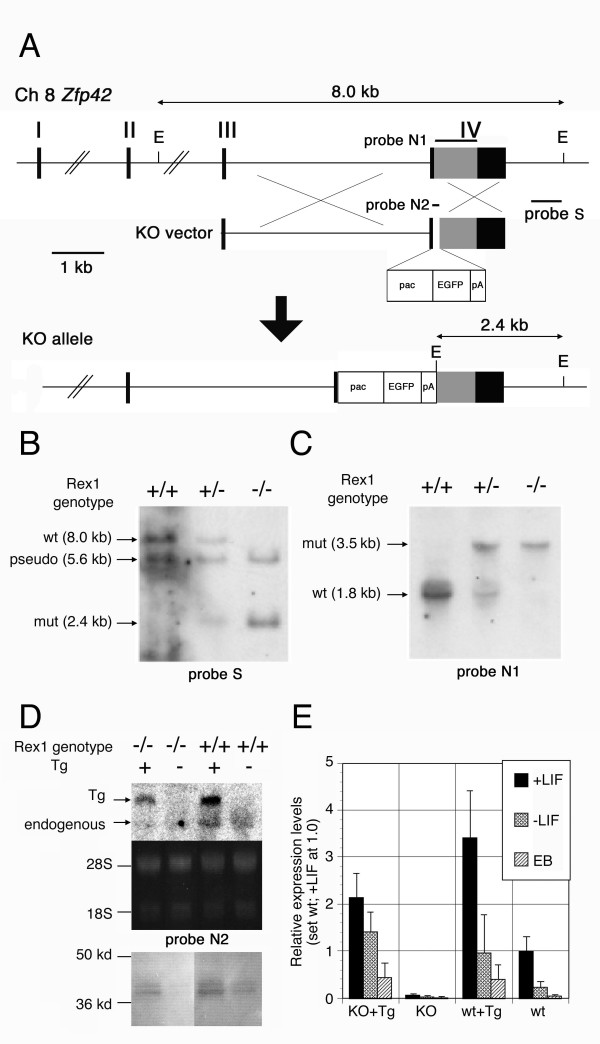
**Generation of ES cells with various *Rex1 *genotypes**. **A**. Strategy for generation of *Rex1*-KO ES cells. The schematic maps of the *Rex1 *allele (top), the KO vector carrying the floxed pacEGFP-pA cassette (middle), and the KO allele generated by homologous recombination (bottom) were shown in scale. The EcoRI sites (E) provide the polymorphism between the wild-type and mutant alleles, 8.0 kb and 2.4 kb, respectively, on southern blot analysis using the indicated 3' external probe. **B**. Southern blot hybridization of wild-type (+/+), *Rex1 *heterozygous (+/-) and homozygous (-/-) ES cells using the EcoRI digestion and the 3' external probe. The expected sizes of wild-type (wt) and mutant (mut) bands were detected. The 5.6 kb fragment corresponds to the polymorphism of the *Rex1 *pseudogene on chromosome 15 reported previously as well as found in the mouse genome data. **C**. Northern blot analysis of *Rex1 *expression in wild-type (+/+), *Rex1 *heterozygous (+/-) and homozygous (-/-) ES cells. The *Rex1 *cDNA probe detects 1.8 kb mRNA from the wild-type allele and 3.5 kb mRNA, which is generated by inefficient function of the polyA addition signal in the pacEGFP-pA cassette, from the mutant allele. The *Rex1 *KO ES cells lack the wild-type transcript. **D**. Northern and western blot analysis of wild-type (+/+) and *Rex1 *KO (-/-) ES cells with the *Rex1 *transgene (Tg:+) or the empty vector (Tg:-). The 2.7 kb transcripts from the transgene were detected with or without the 2.2 kb endogenous transcripts in Northern blot with the *Rex1 *cDNA probe (top), in which equal loading of total RNA was confirmed by ethidium bromide staining of 28S and 18S ribosomal RNAs (middle). Western blot using anti-Rex1 antisera detects ~38 kd band in wild-type, wild-type+Tg and Rex1 KO+Tg lanes but not *Rex1 *KO lane (bottom), confirming the proper production of Rex1 protein from Tg. **E**. QPCR analysis of *Rex1 *expression in undifferentiated (+LIF) and differentiated (-LIF and EB) ES cells with various *Rex1 *genotypes. Three independent clones with each genotypes were cultured with or without LIF for 4 days or for formation of EBs for 5 days, analyzed separately with normalization by the amount of *Gapdh*, and plotted with standard deviation against the expression level in undifferentiated wild-type ES cells (wt) cultured with LIF, set as 1.0. The primer pair was set in the region deleted in the KO allele.

To analyze the precise function of *Rex1 *in the maintenance of pluripotency, we generated a series of genetically-engineered ES cell lines for its gain- and loss-of function analyses. For loss-of-function analysis, we disrupted the endogenous *Rex1 *allele by conventional gene targeting via homologous recombination in ES cells (Fig. 1A). The knock-out (KO) allele should be a functionally null allele because the first 100 bp of the open reading frame in the exon 4 including the start codon was replaced by the *pacEGFP *chimeric gene cassette containing the puromycin-resistant gene (*pac*) and the green fluorescent protein (*Egfp*) cDNA. Interestingly, all of the puromycin-resistant clones obtained by transfection of this KO vector carried the correctly targeted alleles. One of the *Rex1*+/- ES cell line (RKPG9) was cultured with high-dose puromycin to obtain the *Rex1*-/- ES cell lines generated via spontaneous gene conversion. As the result, multiple *Rex1*-/- ES cell lines were established with extremely high efficiency (4 of 4 clones obtained after the selection were homozygous for *Rex1 *KO allele). Correct targeting events were confirmed by the loss of the polymorphic signature of the wild-type allele on the southern blot analysis of the genomic DNA (Fig. 1B), in which the 5.6 kb fragment corresponds to the *Rex1 *pseudogene on chromosome 15 reported previously as well as found in the mouse genome data [22]. Northern blot revealed the loss of the transcript derived from the wild-type allele in *Rex1*-/- ES cells, which express the large transcripts composed by the truncated *Rex1 *and *pacEGFP *(Fig. 1C). *Rex1-/- *ES cells were also established by introduction of the second knockout vector carrying the hygromycin-resistant gene as a selection marker into *Rex1+/- *ES cells with *pacEGFP *followed by the selection with hygromycin B. Genotyping of 5 drug-resistant clones revealed that 2 clones were *Rex1+/- *and 3 clones were *Rex1-/-*, indicating that Rex1-/- ES cells were able to be established without selection pressure (data not shown). Easy isolation of the Rex1-/- ES cells suggested that the *Rex1 *function is not essential for self-renewal of ES cells.

For gain-of-function assay for *Rex1*, the *Rex1 *cDNA isolated from ES cell RNA by RT-PCR was inserted into the expression vector pCAG-IZ [23] and introduced into the wild-type and the *Rex1*-/-ES cells. Since the strong activity of the CAG expression unit [24], the bi-cistronic design of the expression vector with *internal ribosome entry site *(*IRES*) [25] and the character of the zeocin selection system, in which high level expression of the zeocin-resistant gene (*zeo*) is required for the host cells to survive in the medium containing 40 μg/ml of zeocin [26], all of the resulting transfectants express *Rex1 *cDNA at comparative or higher level to that of the endogenous *Rex1 *gene in the wild-type ES cells. Since the efficiency to obtain the zeocin-resistant clones was not different between wild-type and *Rex1*-/-ES cells, the over-expression might not affect self-renewal of ES cells (data not shown).

As the results of these gene manipulations, we established ES cell lines with four different genotypes for *Rex1*; ES cells carrying the wild-type *Rex1 *alleles and the empty CAG-IZ vector (wt), the wild-type *Rex1 *alleles and the *Rex1 *transgene (wt-Tg), the *Rex1*-/- alleles and the empty vector (KO), and the *Rex1*-/- alleles and the *Rex1 *transgene (KO-Tg). Since the *Rex1*-Tg express *Rex1 *at twice levels of the endogenous *Rex1*, the wt-Tg ES cell lines express *Rex1 *at three times more than the wild-type ES cells as the sum of endogenous and exogenous transcripts, whereas the KO-Tg lines express at the twice levels of the wt ES cells (Fig. 1D, E). Both Tg ES cells express Rex1 at the half level of the wt ES cells from the constitutively-active transgene irrespective to the culture condition, whereas the endogenous *Rex1 *gene is down-regulated immediately after induction of differentiation by either withdrawal of leukemia inhibitory factor (LIF) or formation of embryoid bodies (EBs) (Fig. 1E).

### Minor effects of *Rex1 *on the pluripotency-associated transcriptome

**Figure 2:**
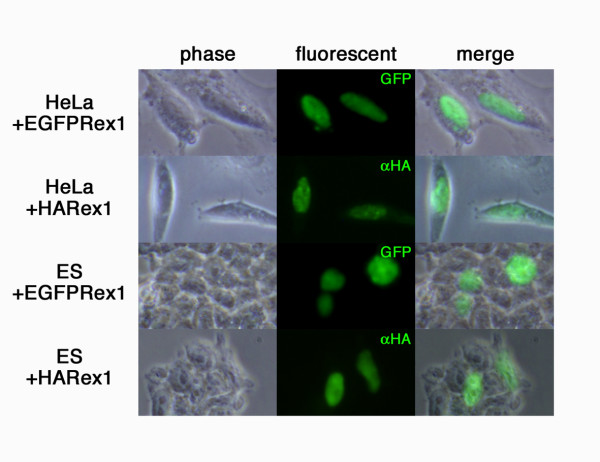
**Nuclear localization of Rex1**. The expression vectors of EGFP-tagged or HA-tagged *Rex1 *were transiently transfected into HeLa and ES cells, and the localization of these transgene products were detected by fluorescent microscopy directly (for EGFPRex1) or after immunostaining for HA-tag (for HARex1). Phase contrast (left), fluorescent (middle) and their merged image (right) were shown for each transfectants. The fluorescent signals were localized in nuclei in both HeLa and ES cells for both chimeric Rex1 proteins.

**Figure 3:**
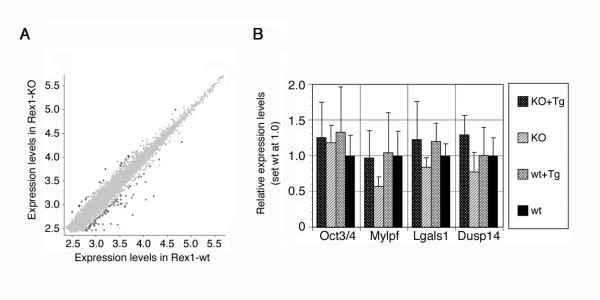
**Gene expression profile in *Rex1 *KO ES cells**. **A**. DNA microarray analysis of *Rex1 *KO ES cells. Scatter-plot of log-ratios of relative expression levels were shown for wild-type (EB5) versus *Rex1 *KO (HP3) ES cells. **B**. QPCR analyses of expressions of the putative Rex1 target genes. Three independent clones with each genotypes were cultured with LIF for 4 days, analyzed separately with normalization by the amount of *Gapdh*, and plotted with standard deviation against the expression level in undifferentiated wild-type ES cells (wt), set as 1.0.

First we tested *Rex1*-dependent transcription in ES cells since the Rex1 protein may function as a transcription factor like its most homologous protein Yy1. Indeed, we confirmed that the chimeric Rex1 proteins with Egfp-tag and HA-tag localize in nuclei (Fig. 2). One of the important functions of Yy1 is mediated by the interaction with the polycomb related complexes (PRCs) [27], and it was reported that Yy1 directly interact to PRCs and recruit them to the targets in sequence-specific manner [28]. Recently, the functional importance of PRCs in ES cells to repress differentiation-related genes has been disclosed [1]. Gene expression profiles were qualitatively examined by the microarray analyses of two *Rex1*-/- ES cells (HP3 and HP4), and one wild-type ES cells (EB5), revealing that very few genes showed significant differences in their expression levels between wild-type and *Rex1*-/- ES cells (Fig. 3A). A pair-wise comparison of *Rex1+/+ *ES cells (EB5) and *Rex1-/- *ES cells (HP3) (false discovery rate (FDR) < 0.05, gene expression difference > 2-fold) showed only 116 genes whose expression levels were significantly different. Among them, 30 genes were up-regulated in *Rex1*-/- ES cells (Additional file 1), whereas 86 genes were down-regulated (Additional file 2). To verify the relationship between the expressions of these genes and the *Rex1 *genotypes, we quantified the expression levels in each three wt, wt+Tg, KO and KO+Tg ES cell lines by quantitative RT-PCR (QPCR). As a result, 3 genes (*Mylpf, Lgals1 *and *Dusp14*) were identified as putative Rex1 target genes since their expressions were down-regulated in KO ES cells, which were restored in KO-Tg ES cells (Fig. 3B). These data suggested that Rex1 may functions as a transcription factor although its impact on the maintenance of the pluripotency-assocoated transcriptome is very faint.

### *Rex1 *does not involve in the maintenance of pluripotency in ES cells

**Figure 4:**
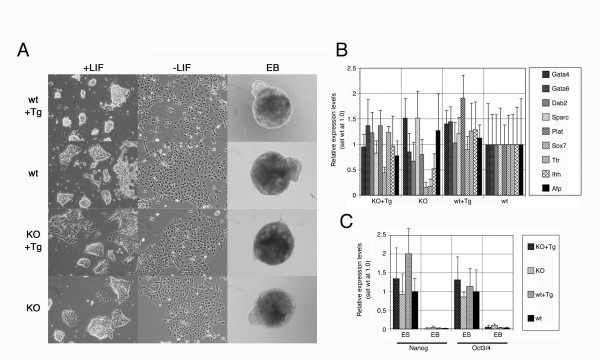
**Differentiation of ES cells with various *Rex1 *genotypes**. **A**. Photomicrographs of colonies at 4 days cultured with LIF (left) or without LIF (middle), or EBs at 5 days (right) derived from representative ES cells with indicated genotypes for Rex1. Scale bar is 100 μm. **B**. QPCR analyses of the endoderm marker genes in EBs derived from ES cells with various Rex1 genotypes. Three independent clones with each genotypes were cultured for formation of EBs for 5 days, analyzed separately with normalization by the amount of *Gapdh*, and plotted with standard deviation against the expression level in wild-type ES cells-derived EBs, set as 1.0. **C**. QPCR analyses of the stem cell marker genes in ES cells and EBs with various *Rex1 *genotypes. Three independent clones with each genotypes were cultured for 4 days with LIF or for formation of EBs for 5 days, analyzed separately with normalization by the amount of *Gapdh*, and plotted with standard deviation against the expression level in wild-type ES cells, set as 1.0.

Using three independent clones each of the ES cell lines with 4 genotypes shown above (wt, wt-Tg, KO and KO-Tg), we tested the function of *Rex1 *in both dominant and recessive manners in the maintenance of pluripotency in ES cells. All ES cell lines propagated at the comparable growth rate and kept normal morphology (Fig. 4A). Gene expression analysis by QPCR revealed no remarkable change among them for several pluripotency-associated markers such as Oct3/4 and Nanog (Fig. 4C and data not shown). All these ES cells completely undergo morphological differentiation for 5 days after withdrawal of LIF (Fig. 4A) with accompanying the up-regulation of a set of primitive endoderm markers such as *Gata4 *[29], *Gata6 *[30] and *Disabled homolog 2 *(*Dab2*) [31] (data not shown). There was no difference in the kinetics of differentiation event although the slight increase of *Nanog *expression was evident in wt-Tg ES cells (Fig. 4C). Therefore, *Rex1 *is incapable to maintain LIF-independent self-renewal either in dominant manner like the case of *Nanog *[8] or the recessive manner like the case of *Mbd3 *[32].

Next we tested the function of *Rex1 *in ES cells in relation to PRCs. It was reported that targeted deletion of the PRC2 component *Eed *resulted in reduced ability of differentiation [33], and that of *Suz12 *caused delayed down-regulation of the pluripotency-assocoated markers during formation of embryoid bodies (EBs) [34]. To test the analogous function of *Rex1*, we generated EBs from wt, wt-Tg, KO and KO-Tg ES cells and analyzed the expression pattern of the various marker genes. The growth and morphology of EBs were indistinguishable among these ES cells (Fig. 4A), indicating that the differentiation ability of KO ES cells was obviously unaffected and persistent expression of Rex1 from the constitutively-active Tg does not affect differentiation event in this context. Moreover, induction of the primitive endoderm markers *Gata4*, *Gata6 *and *Dab2 *occurred normally in these EBs irrespective to their *Rex1 *genotypes (Fig. 4B), and the down-regulation of the pluripotency-assocoated markers *Oct3/4 *and *Nanog *was also unaffected (Fig. 4C). These data as well as the gene expression profile suggested that *Rex1 *function does not involve in global PRC recruitment in ES cells.

### Aberrant induction of some visceral endoderm markers in the absence of *Rex1*

According to the previous analysis of *Rex1*-/- F9 EC cells, *Rex1 *might involve in differentiation of visceral endoderm [20]. In the case of ES cells, visceral endoderm is efficiently imaged on the surface of EBs [35]. Therefore, we generated EBs from wt, wt-Tg, KO and KO-Tg ES cells and analyzed the induction of extraembryonic endoderm marker genes. The growth and morphology of EBs were indistinguishable among these ES cells (Fig. 4A). However, the induction of two visceral endoderm marker genes, *Transthyretin *(*Ttr*) [35] and *Indian hedgehog *(*Ihh*) [36], were reduced in EBs derived from KO ES cells, which were restored in KO-Tg ES-derived EBs, indicating that this phenotype is depend on the expression of *Rex1 *(Fig. 4B). Interestingly, the induction level of the Sry-related transcription factor *Sox7*, which also expresses in visceral endoderm in vivo [37], was also reduced in KO ES-derived EBs and restored in KO-Tg ES-derived EBs, suggesting its involvement in visceral endoderm differentiation. However, the visceral endoderm differentiation might not completely perturbed in the absence of *Rex1 *because *Alphafetoprotein *(*Afp*), which is an archetypal marker for visceral endoderm [38], was normally induced in EBs derived from KO ES cells (Fig. 4B). In contrast, the parietal endoderm markers *Sparc *(*secreted acidic cysteine rich glycoprotein*) [39] and *Plat *(*tPA; plasminogen activator, tissue*) [40], were equally induced in EBs irrespective to their genotypes for *Rex1 *(Fig. 4B). These data indicated that *Rex1 *function might specifically involve in the differentiation of visceral endoderm.

### *Rex1 *function in vivo

**Figure 5:**
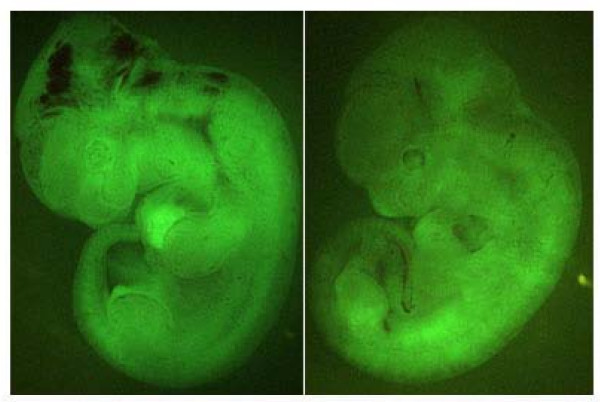
**Chimeric embryos derived from *Rex1 *KO ES cells**. When HP4-EGFP ES cells, which were homozygotes for the mutant *Rex1 *allele and marked by the constitutively-active *Egfp *transgene, were injected into blastocysts, the embryos developed to chimeras at 12.5 *dpc *in which widespread contributions of GFP-positive cells were observed in fluorescent microscopic observation.

To fully evaluate the differentiation ability of the *Rex1*-/- ES cells, we labeled these cells by introduction of the constitutively-active *Egfp *transgene (CAG-*Egfp*-IZ) and injected them into blastocysts followed by transplantation in uteri of pseudo-pregnant mice to generate chimeric embryos. As a result, we obtained the embryos with widespread contribution of the fluorescent cells derived from the *Rex1*-/- ES cells, indicating that these ES cells were pluripotent (Fig. 5).

In the chimeric embryos, all extra-embryonic tissues were derived from the host blastocysts, so the deficiency of visceral endoderm formation of the *Rex1*-/- cells in development should be uncovered. To test this point, we generated the mice carrying the null allele for *Rex1 *by the mating of the male chimeras generated from the heterozygote ES cells with the wild-type female mice. The resulting heterozygotes were obviously normal and healthy, and their intercross resulted in generation of the homozygotes for the *Rex1*-null allele. Although the ratios of the heterozygotes and homozygotes were smaller than the expected mendelian ratios in the offsprings at 8 weeks (Table 1), both homozygous males and females were fertile and showed no morphological abnormalities. Indeed, the homozygous offsprings were obtained by the intercross of the homozygotes, indicating that the *Rex1 *gene show neither the maternal effect nor the critical role in the development of the extraembryonic tissues including visceral endoderm. When we analyzed genotypes of embryos at 17.5 dpc, no significant reduction of heterozygotes and homozygotes was observed (Table 2), suggesting that the loss of embryos with these genotypes occurred peri- or post-natal period. Although the reason of this phenomenon is still unclear, these data indicated that the *Rex1 *function is dispensable for the maintenance of pluripotency in the early developmental period as well as the germ cell development in the mid and late developmental periods.

**Table 1:** Genotype analysis of progeny resulting from crosses of *Rex1 *+/- mice

	No. of mice with indicated genotype (% of total)			
	+/+	+/-	-/-	Total No. of mice

Female	21	26	5	52
Male	22	23	9	54

Total	43 (40.6)	49 (46.2)	14 (13.2)	106 (100)

**Table 2:** Genotype analysis of fetus at 17.5 *dpc *resulting from crosses of *Rex1 *+/- mice

No. of mice with indicated genotype (% of total No)			
+/+	+/-	-/-	Total No. of mice
8	18	9	35
(23)	(51)	(26)	(100)

## Discussion

Cell-type-specific gene expression is one of the important landmarks of gene function in development. In ES cells, microarray analysis, SAGE and in silico subtraction of EST database have been identified several genes specifically expressing in undifferentiated ES cells. Among them, *Rex1 *is one of the oldest marker genes of undifferentiated pluripotent stem cells first reported on 1989 [14]. Since then, the function of *Rex1 *has been kept as a mystery, but here we finally revealed its dispensability to maintain cellular pluripotency.

*Rex1 *was first identified as the gene which expression is repressed by RA in F9 EC cells. A deletion of *Rex1 *from F9 cells resulted in the loss of ability to differentiate into visceral endoderm induced by RA and cyclic AMP analogs without no phenotypical change in their undifferentiated state [20], which is consistent with our result shown here. In contrast, it was recently reported that RNAi-mediated silencing of *Rex1 *in ES cells prevents self-renewal [21]. The controversy between the phenotypes of gene-targeting and gene-silencing might be derived from the different kinetics of silencing or the strong bias of the selection of homozygous mutant ES cells that allow establishment of adopted ES cells for the absence of the particular gene function. Although both might be the case for *Rex1*, neither ES cells nor mouse embryos show any phenotype in the maintenance of pluripotency, suggesting that the imperfect target specificity of gene-silencing by RNAi might be most responsible to give the discrepancy in this case since the expression vector to generate large double-strand RNA was applied in the above report.

Why does the function of *Rex1 *revealed here look so minor although the previous reports suggested its importance in ES cells as the common target of the pluripotency-associated transcription factors Oct3/4, Sox2 and Nanog [22,41-43] as well as the component of the protein-interaction network [44]? One simple explanation is functional redundancy between *Rex1 *and its related gene(s) present in the genome. *Rex1 *encodes the C2H2-type zinc-finger whose sequence is most similar to the transcription factor Yy1 [45]. *Yy1 *widely expresses in various tissues in embryos and adults and its function is essential for peri-implantation development [46]. Since *Rex1 *express in inner cell mass of the blastocyst-stage embryos and its expression is down-regulated after implantation [16,47], the pre-implantation development of *Yy1*-null embryos might be supported by the overlapped function of *Rex1 *and its down-regulation after implantation might cause their lethality. The function of *Yy1 *is pleiotropic [15] and one of them is the function to recruit PRCs to the specific target sequences to repress the transcription of the target genes [27,28]. Although the several reports showed the function of the polycomb complex for the maintenance of pluripotency in ES cells [1], the function of *Yy1 *in pluripotent stem cells has yet been confirmed. Since *Yy1 *expresses in ES cells, it may mask the phenotype of the *Rex1*-/- ES cells by the functional redundancy. It was recently reported that *Rex1 *is a pseudogene generated from *Yy1 *by retrotransposition [45]. The zinc-finger domain of Yy1 is encoded by 3 exons whereas that of Rex1 is encoded in a single exon, and *Yy1 *is evolutionally conserved in all vertebrates, whereas *Rex1 *is found only in placental mammals. This hypothesis suggests that *Yy1 *occupies the major role in the redundant function to *Rex1 *and *vice versa*. The specific function of *Rex1 *to induce particular markers in visceral endoderm might be conducted by its different preference of the target sequence from Yy1 [45] or down-regulation of *Yy1 *in this cell type.

The most prominent phenotype of the *Rex1 *deficiency is the poor induction of a subset of visceral endoderm marker genes. In the case of F9 EC cells, the null mutation for *Rex1 *completely prevent differentiation of visceral endoderm induced by RA and cAMP analogs, which was evaluated by the complete loss of induction of one of the visceral endoderm markers, *Afp*. In contrast, in ES cells, the suspension culture allows induction of visceral endoderm without any small molecule inducers, and the loss of *Rex1 *affects the induction of *Ttr *and *Ihh*, but not *Afp*. How can the *Rex1 *deficiency modulate the expression of the visceral endoderm marker genes although its expression is tightly restricted in pluripotent stem cells and down-regulated during their differentiation (Fig. 1E and [16,47])? One possible explanation is that the gene(s) expressing in the pluripotent stem cells under the control of Rex1 might induce the gene(s) in the visceral endoderm adjacent to them in EBs like the case of *Fibroblast growth factor-4 *(*Fgf4*), which is produced by the pluripotent stem cells and acts as a paracrine growth factor for the extra-embryonic endoderm cells and the trophectoderm cells [48]. However, the functional significance of the reduced levels of these visceral endoderm markers associated to the *Rex1 *deficiency should be very minor because the *Rex1*-null embryos are absolutely capable to develop normally in early embryogenesis.

It is a surprise that this is the first report of the *Rex1 *knockout mice because this gene was discovered 18 years ago, has been regarded as an important marker of pluripotent stem cells, and the generation of the heterozygous ES cell line via gene-trap was reported in 1992 [49]. The cell-type-specific expressions of genes have been regarded as good landmarks of their functions. Indeed, *Oct3/4 *and *Nanog *show highly specific expression in pluripotent stem cells in vitro as well as in vivo and exhibit essential function to maintain pluripotency. However, there are several exceptions of this relationship in the genes specifically express in pluripotent stem cells. In the cases of *Fbxo15*, *Sox15 *and *Dppa5/Esg1*, the null mutant embryos for them show no abnormality and the null ES cells are capable to be established and maintained although their expression is as tightly restricted in pluripotent stem cells as the case of *Oct3/4 *[50-52]. The discrepancy between the specific expression and the function in pluripotent stem cells might due to the global down-regulation of gene expression during differentiation of pluripotent stem cells via epigenetic mechanism. It was discovered that the epigenetic repression of gene expression is quite loose in the pluripotent stem cells, which is established during their differentiation [2]. In addition, the cell-cycle regulation is also dramatically altered during differentiation since the S-phase is enriched in ES cells whereas the G1 phase is predominant in differentiated cells [53]. Therefore, no specific transcriptional regulation might be required for genes to exhibit the stem-cell-specific expression pattern especially if their expressions depend on the cell-cycle. Indeed, in the case of *Rex1*, the octamer motif for the binding of Oct3/4 identified in the mouse *Rex1 *promoter [22,41] is not conserved in the human *Rex1 *promoter based on the Ensembl database search, although the previous reports confirmed that both are capable to direct stem-cell-specific transgene expression [17], suggesting that the Oct3/4-independent regulation might be critical for its stem-cell-specific expression.

This is not the end of the story of *Rex1*, however. The intercross of the *Rex1 *heterozygotes revealed mild reduction of the homozygous pups between 17.5 dpc and 8 weeks after the birth. Although the reason of this lethality with low penetrance is completely mysterious since the expression of *Rex1 *is only detectable in the germ cells at the late-stage embryos and the pups, *Rex1 *should have some function apart from the pluripotent stem cells, which is out of our scope of the research. We hope the *Rex1 *mutant mice strain we generated will be applied for the further studies to unlock the mystery of the *Rex1 *function in the differentiated cell lineage.

## Conclusion

We showed that Rex1 function is dispensable for self-renewal of mouse ES cells. Although its possible function in pluripotent cells in particular developmental context has not been ruled out completely, its function is not required to maintain pluripotency in its conventional meaning.

## Methods

### Cell culture and transfection

E14tg2a [54] and its derivatives appear in this paper were cultured in the absence of feeder cells in Glasgow minimal essential medium (GMEM) supplemented with 10% fetal calf serum, 1 mM sodium pyruvate, 1× nonessential amino acids, 10^-4 ^M 2-mercaptoethanol, and 1000 U of leukemia inhibitory factor (LIF) per ml on gelatin-coated dishes. For the transfection, 10^7 ^ES cells were electroporated with 50 μg of linealized plasmid DNA at 800 V and 3 μF in a 0.4-cm cuvette using a Gene Pulser (Bio-Rad) followed by the culture with appropriate selection drugs (1.5 μg/ml of puromycin [Sigma] or 40 μg/ml of zeocin [Invivogen]) for 7 to 10 days. For the selection of *Rex1*-/- ES cells, the heterozygous mutant ES cells were cultured in the presence of 9 μg/ml of puromycin in the first 3 days. HeLa cells were cultured in GMEM supplemented with 10% FCS. For transient expression of *EGFP-Rex1 *and *HA-Rex1*, 2 μg of plasmid DNA was transfected into 3 × 10^4 ^cells using Lipofectoamine 2000 (Invitrogen).

For induction of differentiation, 2 × 10^4 ^cells were seeded in 60-mm dish in the presence or absence of LIF and cultured for 4 days, or 300 cells were cultured in 15 μl of hanging drop in the ES culture medium without LIF for 5 days to generate embryoid body (EB).

### Construction of KO vector and expression vectors

For generation of *Rex1*-KO vector, the genomic DNA fragment for 5' and 3' homology arms were amplified from the E14tg2a genomic DNA using the primer pairs 5'-CAACTTTTTATTTCCCATTCACAGCTC-3' and 5'-TCTTAGCTGCTTCCTTGAACAATGCC-3', and 5'-AAACTAGTGAATTCCAGAATACCAGAGTGG-3' and 5'-AGCGGCCGCTTCAATAGCACATATAGTAAG-3', respectively. The 4.2 kb of 5' homology arm was sub-cloned into the EcoRV site of pBlKS(-) then excised by SalI and SpeI, and the 3' homology arm was digested by SpeI and NotI. These DNA fragments were subcloned into SalI and NotI of pBlKS(-), resulting pRex1 5'+3'. The pacEGFP-pA cassette carrying the fusion gene of *puromycin acetyltransferase *(*pac*) and *Egfp *(HN, unpublished) was inserted into HindIII-BamHI between the *loxP *sites of pBS246 (Life Technologies), and the SpeI-EcoRI fragment with loxP-pacEGFP-pA-loxP was inserted into SpeI-EcoRI of pRex1 5'+3', resulting pRex1-KO. The 300 bp fragment of genomic DNA at 3' external region of the Rex1 KO vector, which was amplified using the primers 5'-TGGGGACTTTTGCATACGGCAG-3' and 5'-GAACTCATTTCTAGTGTTTTATTTTC-3' and subcloned into the EcoRV site of pBlKS(-), was used as a probe for southern blot analysis of the homologous recombinants.

The *Rex1 *expression vector was constructed by inserting the *Rex1 *cDNA amplified from cDNA of E14tg2a ES cells using the primers 5'-GACATCATGAATGAACAAAAAATG-3' and 5'-CCTTCAGCATTTCTTCCCTG-3' into the BstXI sites of pCAG-IZ [26] using the BstXI adaptors. For generation of the expression vectors of *EGFP-Rex1 *and *HA-Rex1*, the BspHI-NotI fragment of pCAG-Rex1-IZ was excised and introduced into NcoI-NotI of pCAG-EGFPOct3-IP and pCAG-HAOct3-IP [23], respectively.

### Immunoblotting and immunohistochemistry

The whole cell lysates were fractionated on sodium dodecyl sulfate (SDS)-10% polyacrylamide gel and electroblotted onto a polyvinylidene difluoride membrane. After treatment in blocking buffer (1× TTBS [10 mM Tris HCl {pH 7.4}, 137 mM NaCl, 2.7 mM KCl, 0.1% Tween 20] plus 3% skimmed milk), membrane was probed with the rabbit anti-Rex1 antisera raised against the GST-Rex1 fusion protein and then horseradish peroxidase-conjugated anti-rabbit immunoglobulin G antibody and developed using ECL reagents (GE Healthcare). For the detection of HA-Rex1 in the transient transfectants, 24 hours after transfection of pCAG-HA-Rex1-IP, the cells were fixed by 4% paraformaldehyde in phosphate-buffered saline (PBS) for 30 minutes at 4°C and then permealized by 0.2% Triton X-100 in PBS for 15 minutes at room temperature. After brief washing with PBS followed by blocking by 2% FCS in PBS, the cells were stained with mouse anti-HA antibody (clone 262 K; Cell Signaling Technology) and then Alexa Fluor 488-conjugated goat anti-mouse antibody (Molecular Probes). The fluorescent images were captured with an IX51 microscope (Olympus) and DP70 digital camera (Olympus).

### Northern blot and Quantitative PCR

Total RNA was prepared using TRIzol reagent (Invitrogen) or QUICKGene RNA cultured cell kit (FUJIFILM) according to the manufactures' instructions. For Northern blot analysis, 4 μg of total RNA was analyzed by non-radioactive filter hybridization (Gene Image; GE Healthcare) with either the *Rex1 *cDNA probe (probe N1) or the *Rex1 *cDNA fragment probe hybridize to the region deleted in the KO allele (for probe N2). First strand cDNA was synthesized from 1 μg of total RNA in 40 μl of the reaction mixture containing oligo-dT primers using a ReverTra Ace first strand synthesis kit (Toyobo). Real-time PCR was performed with the ExTaq cyber green supermix (Takara) using an iCycler System (Bio-Rad). The amount of target RNA was determined from the appropriate standard curve, and was normalized relative to the amount of *Gapdh *mRNA. Sequences of primer pairs were described previously [55] as well as shown below.

*Rex1*; forward 5'-ttgcctcgtcttgctttagg-3' and reverse 5'-aaaatgaatgaacaaatgaagaaaa-3', *Mylpf*; forward 5'-gcccccaggagatctaagac-3' and reverse 5'-ccactggcttccttcatcat-3', *Lgals1*; forward 5'-ctctcgggtggagtcttctg-3' and reverse 5'-gcgaggattgaagtgtaggc-3', *Dusp14*; forward 5'-gaagatcaagggcagctcag-3' and reverse 5'-tcccagggcacactaatttc-3'.

### Microarray analysis

DNA microarray analyses were performed as described previously [56], using an NIA Mouse 22 K Microarray v1.1 (manufactured by Agilent Technologies: #11472, G4120A), which contained the genes listed at the National Institute of Aging mouse cDNA project web site . Briefly, 5 μg of total RNA was transcribed into double-strand T7 RNA polymerase-tagged cDNA and amplified into single-stranded, fluorescence-tagged cRNA by T7 polymerase. The samples for wild-type (EB5) and *Rex1*-/- (HP3, HP4) ES cells were hybridized against a universal reference RNA at 60°C on the DNA microarrays. After washing, microarrays were scanned with an Agilent DNA Microarray Scanner. Microarray results were analyzed using NIA Array Analysis Software [57]. Complete array data will be available on the GEO (NCBI) website.

### Production of chimeric embryos and mice

To visualize the *in vivo *contribution of *Rex1*-/- ES cells, HP4 ES cells were transfected with the constitutive Egfp expression vector (pCAG-Egfp-IZ), resulting establishment of HP4-EGFP ES cells. To obtain chimeric embryos, HP4-EGFP ES cells were injected into C57Bl/6J blastocysts, followed by transfer into the uteri of pseudopregnant ICR mice. Embryos were dissected at 12.5 *dpc *and fluorescent signals were detected using an Olympus SZX12 fluorescent dissecting microscope and captured with an Olympus DP70 digital Camera.

To establish the mouse strain carrying the mutated *Rex1 *allele, heterozygous ES cells were injected into C57Bl/6J blastocysts to generate chimeric mice. Male germline chimera was crossed with C57Bl/6J females to obtain heterozygous litters, in which the transmission of the mutated *Rex1 *allele was monitored by southern blot using the 3' external probe. These heterozygotes were intercrossed for generation of homozygotes, and the homozygotes were mated to confirm their fertilities.

## Authors' contributions

SM and HN carried out experiments with the help of RY and KT. SO performed mouse embryo manipulations, and MSHK performed microarray analysis. HN conceived the study, reviewed and analyzed all data and drafted the manuscript.

## Supplementary Material

Additional file 1 List of genes up-regulated in Rex1-/- ES cells identified by microarray analysis. Click here for file

Additional file 2 List of genes down-regulated in Rex1-/- ES cells identified by microarray analysis. Click here for file
