# Supplementary material for: Rex1/Zfp42 is dispensable for pluripotency in mouse ES cells
Source: BMC Dev Biol. 2008 Apr 24;8:45. doi: 10.1186/1471-213X-8-45 (PMC2386458; doi:10.1186/1471-213X-8-45)
Supplement: Additional file 1 — List of genes up-regulated in Rex1-/- ES cells identified by microarray analysis. [file 1471-213X-8-45-S1.doc]

Supplemental Table S1. List of genes up-regulated in *Rex1-/-* ES cells

Featureid Mean Mean Log Fold t P FDR Noisy GeneIndex RefSeq GenBank MGI Symbol Annotation

(HP3) (EB5) Ratio Change ‘U’ Cluster Accession Accession

Z00001928-1 3.75 3.2 0.547 3.52 16.49 0 0 0 U012539 NM_013529.1 NM_013529.1 MGI:1338883 Gfpt2 glutamine fructose-6-phosphate transaminase 2

Z00014161-1 3.9 3.44 0.457 2.86 7.81 0 0 1 U022704 "H3064F11-3 NIA Mouse 15K cDNA Clone Set Mus musculus cDNA

clone H3064F11 3', mRNA sequence"

Z00002958-1 3.47 3.09 0.386 2.43 9.65 0 0 0 U035955 XM_980979.1 LOC624168 similar to zinc finger protein of the cerebellum 5

Z00015666-1 3.07 2.7 0.366 2.32 7.58 0 0 0 Intronic in U042659

Z00002972-1 4.34 3.98 0.365 2.32 7.9 0 0 1 U014381 AK122380.1 MGI:1929461 Lphn1 latrophilin 1

Z00005932-1 2.86 2.5 0.358 2.28 10.97 0 0 0 U008764 NM_145587.2 NM_145587.2 MGI:2135937 Sbk1 SH3-binding kinase 1

Z00009936-1 3.15 2.8 0.355 2.27 11.71 0 0 0 U023053 NM_001033452.2 NM_001033452.2 MGI:3584519 Gm1967 "gene model 1967, (NCBI)"

Z00018770-1 3.98 3.63 0.354 2.26 6.75 0 0 1 Intronic in U068788

Z00004978-1 4.03 3.68 0.35 2.24 9.9 0 0 0 U035481 NM_144841.2 NM_144841.2 MGI:97451 Otx2 orthodenticle homolog 2 (Drosophila)

Z00002447-1 3.51 3.16 0.347 2.23 11.76 0 0 0 U035424 NM_130878.2 NM_130878.2 MGI:2157782 Pcdh21 protocadherin 21

Z00005303-1 3.88 3.54 0.34 2.19 17.01 0 0 0 U032336 NM_010296.2 NM_010296.2 MGI:95727 Gli1 GLI-Kruppel family member GLI1

Z00020371-1 4.1 3.77 0.34 2.19 7.95 0 0 1 Intronic in U040642

Z00014969-1 3.17 2.84 0.337 2.17 6.9 0 0 1 U032518 NM_172496.2 NM_172496.2 MGI:105056 Cobl cordon-bleu

Z00002317-1 2.99 2.65 0.334 2.16 9.2 0 0 0 U360008 "H8243D01-3 NIA Mouse Unique Gene Set Version 2 Mus musculus

cDNA clone H8243D01 3', mRNA sequence"

Z00002546-1 3.46 3.13 0.329 2.13 6.46 0 0 1 U034899 AB214501.1 MGI:105373 Ptch1 patched homolog 1

Z00010623-1 3.83 3.5 0.329 2.13 5.61 0 0 1

Z00020280-1 3.95 3.62 0.328 2.13 5.85 0 0 1 U032643 NM_023146.1 NM_023146.1 MGI:1929706 Ranbp17 RAN binding protein 17

Z00021487-1 4.99 4.67 0.325 2.11 14.95 0 0 0 AK155982.1 MGI:1202886 Gtl2 "GTL2, imprinted maternally expressed untranslated mRNA"

Z00003857-1 4.1 3.77 0.325 2.11 5.5 0 0 1 Intronic in U024970

Z00021744-1 3.11 2.79 0.324 2.11 12.15 0 0 0 U051294 AK019066.1 MGI:1925736 #2210414L08Rik RIKEN cDNA 2210414L08 gene

Z00017514-1 3.44 3.12 0.324 2.11 4.66 0 0 1 U350222 "K0637B11-3 NIA Mouse Hematopoietic Stem Cell (Lin-/c-Kit-/Sca-

1+) cDNA Library (Long) Mus musculus cDNA clone NIA:K0637B11

IMAGE:30072310 3', mRNA sequence"

Z00020474-1 3.45 3.13 0.324 2.11 5.17 0 0 1 U045682 "H4029C10-3 NIA Mouse 7.4K cDNA Clone Set Mus musculus cDNA

clone H4029C10 3', mRNA sequence"

Z00009239-1 3.85 3.53 0.323 2.1 5.24 0 0 1 U035625 "UI-M-AP1-agf-c-10-0-UI.s2 NIH_BMAP_MST_N Mus musculus cDNA

clone UI-M-AP1-agf-c-10-0-UI 3', mRNA sequence"

Z00013901-1 3.95 3.64 0.317 2.07 5.63 0 0 1 U346323 "C86530 Mouse fertilized one-cell-embryo cDNA Mus musculus cDNA

clone J0228C09 3', mRNA sequence"

Z00011743-1 2.96 2.65 0.314 2.06 12.47 0 0 0 U003355 NM_019971.2 NM_019971.2 MGI:1859631 Pdgfc "platelet-derived growth factor, C polypeptide"

Z00011309-1 4.24 3.93 0.312 2.05 8.12 0 0 1 U024508 NM_026672.2 NM_026672.2 MGI:1915562 Gstm7 "glutathione S-transferase, mu 7"

Z00000233-1 4 3.69 0.312 2.05 9.04 0 0 0 U039780 NM_001002272.2 NM_001002272.2 MGI:1928994 Tro trophinin

Z00015341-1 3.9 3.59 0.306 2.02 6.67 0 0 1 AK050667.1 MGI:1346873 Map3k2 mitogen activated protein kinase kinase kinase 2

Z00013986-1 3.25 2.95 0.305 2.02 4.9 0 0 1 U010560 AK163559.1 MGI:88582 Cyp11a1 "cytochrome P450, family 11, subfamily a, polypeptide 1"

Z00004297-1 3.82 3.52 0.303 2.01 17.31 0 0 0 U040947 "C0927D07-3 NIA Mouse 12.5-dpc Male Genital Ridge/Mesonephros

cDNA Library (Long) Mus musculus cDNA clone NIA:C0927D07

IMAGE:30037194 3', mRNA sequence"
