# Supplementary material for: Rex1/Zfp42 is dispensable for pluripotency in mouse ES cells
Source: BMC Dev Biol. 2008 Apr 24;8:45. doi: 10.1186/1471-213X-8-45 (PMC2386458; doi:10.1186/1471-213X-8-45)
Supplement: Additional file 2 — List of genes down-regulated in Rex1-/- ES cells identified by microarray analysis. [file 1471-213X-8-45-S2.doc]

Supplemental Table S2. List of genes down-regulated in *Rex1-/-* ES cells

Featureid Mean Mean Log Fold t P FDR Noisy GeneIndex RefSeq GenBank MGI Symbol Annotation

(HP3) (EB5) Ratio Change ‘U’ Cluster Accession Accession

Z00006684-1 3.1821 4.2117 -1.0296 10.705 56.393 0 0 0 U036682 AK166854.1 MGI:96705 Krt8 keratin 8

Z00010859-1 2.5104 3.4982 -0.9878 9.722 17.837 0 0 1 U016695 NM_010664.1 NM_010664.1 MGI:96692 Krt18 keratin 18

Z00005393-1 2.7853 3.7681 -0.9828 9.611 37.874 0 0 0 U016695 NM_010664.1 NM_010664.1 MGI:96692 Krt18 keratin 18

Z00010216-1 3.1068 4.0428 -0.936 8.629 48.015 0 0 0 U005829 NM_013470.1 NM_013470.1 MGI:1201378 Anxa3 annexin A3

Z00016189-1 2.5793 3.4583 -0.879 7.568 23.271 0 0 0 U002275 NM_011580.3 NM_011580.3 MGI:98737 Thbs1 thrombospondin 1

Z00013224-1 2.5978 3.31259 -0.71479 5.185 26.639 0 0 0 U021280 NM_010212.2 NM_010212.2 MGI:1338762 Fhl2 four and a half LIM domains 2

Z00006307-1 2.4687 3.17169 -0.70299 5.046 26.199 0 0 0 U013334 NM_008471.2 NM_008471.2 MGI:96693 Krt19 keratin 19

Z00019741-1 2.9472 3.6057 -0.6585 4.555 27.826 0 0 0 U030382 NM_009606.1 NM_009606.1 MGI:87902 Acta1 "actin, alpha 1, skeletal muscle"

Z00005095-1 2.9823 3.6124 -0.6301 4.266 33.209 0 0 0 U012007 NM_009344.1 NM_009344.1 MGI:1096880 Phlda1 "pleckstrin homology-like domain, family A, member 1"

Z00000428-1 3.0325 3.64639 -0.61389 4.11 30.694 0 0 0 U038902 NM_010730.1 NM_010730.1 MGI:96819 Anxa1 annexin A1

Z00017989-1 3.1507 3.75809 -0.60739 4.049 23.643 0 0 0 U001654 NM_146120.3 NM_146120.3 MGI:95851 Gsn gelsolin

Z00006260-1 3.2042 3.7871 -0.5829 3.827 25.896 0 0 0 U042728 AK160593.1 MGI:88250 Cald1 caldesmon 1

Z00004736-1 3.4781 4.0444 -0.5663 3.683 31.017 0 0 0 U042728 AK148009.1 MGI:88250 Cald1 caldesmon 1

Z00017323-1 2.5914 3.14869 -0.55729 3.608 11.236 0 0 0 U069402 AF138745.1 MGI:1336196 Tsix "X (inactive)-specific transcript, antisense"

Z00008184-1 3.4758 4.0223 -0.5465 3.519 13.051 0 0 1 U033318 NM_029755.1 NM_029755.1 MGI:1343177 Ndp52 nuclear domain 10 protein 52

Z00004742-1 2.4725 3.00079 -0.52829 3.375 16.277 0 0 0 U029486 NM_010514.2 NM_010514.2 MGI:96434 Igf2 insulin-like growth factor 2

Z00009583-1 3.0259 3.54239 -0.51649 3.284 27.74 0 0 0 U032073 NM_001004363.1 NM_001004363.1 MGI:1925226 Nuak1 "NUAK family, SNF1-like kinase, 1"

Z00001280-1 3.019 3.51679 -0.49779 3.146 22.88 0 0 0 U011036 NM_020520.2 NM_020520.2 MGI:1928738 Slc25a20 "solute carrier family 25 (mitochondrial carnitine/acylcarnitine

translocase), member 20"

Z00014574-1 3.8415 4.3383 -0.4968 3.139 11.753 0 0 1 U003502 NM_011313.2 NM_011313.2 MGI:1339467 S100a6 S100 calcium binding protein A6 (calcyclin)

Z00004852-1 4.3211 4.80439 -0.48329 3.042 34.174 0 0 0 NM_008818.2 NM_008818.2 MGI:97538 Rhox5 reproductive homeobox 5

Z00000336-1 2.9972 3.4615 -0.4643 2.912 21.648 0 0 0 U016689 NM_033073.2 NM_033073.2 MGI:96704 Krt7 keratin 7

Z00010007-1 2.595 3.058 -0.463 2.904 8.443 0 0 0 U006564 NM_007616.2 NM_007616.2 MGI:102709 Cav1 "caveolin, caveolae protein 1"

Z00003373-1 2.4599 2.91279 -0.45289 2.837 14.418 0 0 0 U028843 NM_029614.3 NM_029614.3 MGI:1923703 Prss23 "protease, serine, 23"

Z00002086-1 2.9904 3.4403 -0.4499 2.817 8.95 0 0 1 U010727 AK036172.1 MGI:2444710 Prtg protogenin homolog (Gallus gallus)

Z00018218-1 3.1878 3.63429 -0.44649 2.795 21.126 0 0 0 U032280 NM_178057.2 NM_178057.2 MGI:101761 Hmga2 high mobility group AT-hook 2

Z00016591-1 2.561 2.97379 -0.41279 2.586 6.714 0 0 0 U022807 BC036146.1 MGI:1095418 Tfpi tissue factor pathway inhibitor

Z00018468-1 3.4407 3.8528 -0.4121 2.582 22.571 0 0 0 U022807 NM_011576.1 NM_011576.1 MGI:1095418 Tfpi tissue factor pathway inhibitor

Z00003433-1 3.1095 3.51939 -0.40989 2.569 22.451 0 0 0 U000842 AK169720.1 MGI:88549 Csrp1 cysteine and glycine-rich protein 1

Z00015414-1 2.8508 3.2519 -0.4011 2.518 16.108 0 0 0 U017848 AK031801.1 MGI:1346527 Psmb8 "proteosome (prosome, macropain) subunit, beta type 8 (large

multifunctional peptidase 7)"

Z00019201-1 3.0612 3.46079 -0.39959 2.509 18.767 0 0 0 U025230 NM_007408.3 NM_007408.3 MGI:87920 Adfp adipose differentiation related protein

Z00001000-1 2.9853 3.38479 -0.39949 2.508 18.626 0 0 0 U023945 NM_009673.1 NM_009673.1 MGI:106008 Anxa5 annexin A5

Z00011993-1 2.8112 3.2043 -0.3931 2.472 15.224 0 0 0 U016408 NM_027219.1 NM_027219.1 MGI:1929763 Cdc42ep1 CDC42 effector protein (Rho GTPase binding) 1

Z00006521-1 2.6128 3.00539 -0.39259 2.469 13.71 0 0 0 U043893 NM_001007580.1 NM_001007580.1 MGI:2685630 Gm784 "gene model 784, (NCBI)"

Z00014114-1 2.5687 2.96 -0.3913 2.462 11.327 0 0 0 U022179 NM_009367.1 NM_009367.1 MGI:98726 Tgfb2 "transforming growth factor, beta 2"

Z00003467-1 2.8567 3.2404 -0.3837 2.419 16.614 0 0 0 U016410 NM_009164.2 NM_009164.2 MGI:104603 Sh3bp1 SH3-domain binding protein 1

Z00010026-1 2.8702 3.24789 -0.37769 2.386 15.96 0 0 0 U037990 NM_207654.1 NM_207654.1 MGI:107444 Efna5 ephrin A5

Z00016731-1 2.5921 2.9644 -0.3723 2.356 11.773 0 0 0 U018216 XM_128781.8 MGI:2684937 Lycat lysocardiolipin acyltransferase

Z00000446-1 3.9517 4.3224 -0.3707 2.348 13.216 0 0 0 U016412 NM_008495.2 NM_008495.2 MGI:96777 Lgals1 "lectin, galactose binding, soluble 1"

Z00005188-1 2.7762 3.13819 -0.36199 2.301 10.392 0 0 0 U026967 NM_010228.3 NM_010228.3 MGI:95558 Flt1 FMS-like tyrosine kinase 1

Z00017636-1 2.4961 2.85689 -0.36079 2.295 11.486 0 0 0 U033203 NM_019819.2 NM_019819.2 MGI:1927168 Dusp14 dual specificity phosphatase 14

Z00001443-1 2.626 2.9867 -0.3607 2.294 9.733 0 0 0 U030484 XM_919481.2 MGI:1922973 Amotl1 angiomotin-like 1

Z00017352-1 2.497 2.85679 -0.35979 2.289 7.344 0 0 0 U024487 NM_007651.2 NM_007651.2 MGI:88341 Cd53 CD53 antigen

Z00020495-1 2.4584 2.81419 -0.35579 2.268 10.507 0 0 0

Z00000278-1 2.833 3.18869 -0.35569 2.268 9.916 0 0 0 U006508 NM_130877.2 NM_130877.2 MGI:2157785 Peg10 paternally expressed 10

Z00006634-1 3.6554 4.00839 -0.35299 2.254 20.61 0 0 0 U011306 NM_022032.1 NM_022032.1 MGI:1929938 Perp "PERP, TP53 apoptosis effector"

Z00010868-1 3.5938 3.9442 -0.3504 2.24 20.009 0 0 0 U008793 NM_016754.3 NM_016754.3 MGI:97273 Mylpf "myosin light chain, phosphorylatable, fast skeletal muscle"

Z00019665-1 3.0711 3.42079 -0.34969 2.237 18.098 0 0 0 U032502 NM_008343.2 NM_008343.2 MGI:96438 Igfbp3 insulin-like growth factor binding protein 3

Z00018182-1 2.8281 3.17269 -0.34459 2.211 8.669 0 0 0 U095787 NM_009825.1 NM_009825.1 MGI:88283 Serpinh1 "serine (or cysteine) peptidase inhibitor, clade H, member 1"

Z00016612-1 3.0298 3.3726 -0.3428 2.201 13.145 0 0 0 U022215 NM_198247.1 NM_198247.1 MGI:2443496 Sertad4 SERTA domain containing 4

Z00019839-1 2.5375 2.87809 -0.34059 2.19 6.821 0 0 0 U009285 NM_026840.2 NM_026840.2 MGI:1916047 Pdgfrl platelet-derived growth factor receptor-like

Z00015595-1 2.7335 3.07239 -0.33889 2.182 12.688 0 0 0 U023589 NM_009373.3 NM_009373.3 MGI:98731 Tgm2 "transglutaminase 2, C polypeptide"

Z00006007-1 3.1876 3.5251 -0.3375 2.175 18.303 0 0 0 U003796 NM_010171.2 NM_010171.2 MGI:88381 F3 coagulation factor III

Z00013345-1 3.1186 3.4539 -0.3353 2.164 13.465 0 0 0 U038980 NM_007392.2 NM_007392.2 MGI:87909 Acta2 "actin, alpha 2, smooth muscle, aorta"

Z00012632-1 2.6044 2.93959 -0.33519 2.163 10.394 0 0 0 U000472 AK164360.1 MGI:109282 Speg SPEG complex locus

Z00005309-1 2.8005 3.1354 -0.3349 2.162 11.89 0 0 0 AF195850.1 MGI:1346865 Mapk14 mitogen activated protein kinase 14

Z00016305-1 2.6906 3.02369 -0.33309 2.153 4.616 0 0 1 U019389 NM_008332.2 NM_008332.2 MGI:99449 Ifit2 interferon-induced protein with tetratricopeptide repeats 2

Z00002113-1 2.5397 2.8716 -0.3319 2.147 10.099 0 0 0 U026415 NM_175270.2 NM_175270.2 MGI:1925338 Ankrd56 ankyrin repeat domain 56

Z00008032-1 2.8786 3.20999 -0.33139 2.144 13.76 0 0 0 U001608 NM_145144.1 NM_145144.1 MGI:1919598 2810003C17Rik RIKEN cDNA 2810003C17 gene

Z00018265-1 3.3884 3.7194 -0.331 2.142 19.781 0 0 0 U010696 NM_007585.3 NM_007585.3 MGI:88246 Anxa2 annexin A2

Z00016288-1 3.8085 4.1393 -0.3308 2.141 10.866 0 0 0 Intronic in U001401

Z00019679-1 2.5412 2.8706 -0.3294 2.135 10.705 0 0 0 U021529 AK136875.1 MGI:99454 Irs1 insulin receptor substrate 1

Z00011614-1 3.8785 4.2065 -0.328 2.128 8.724 0 0 1 U001401 NM_011701.3 NM_011701.3 MGI:98932 Vim vimentin

Z00005105-1 2.6466 2.974 -0.3274 2.125 6.165 0 0 0 U016845 NM_011415.2 NM_011415.2 MGI:1096393 Snai2 snail homolog 2 (Drosophila)

Z00000582-1 3.3555 3.68279 -0.32729 2.124 9.501 0 0 0 U358399 #Gm719 "ii44g01.x1 Melton Normalized Mixed Mouse Pancreas 1 N1-MMS1

Mus musculus cDNA clone IMAGE:5944513 3', mRNA sequence"

Z00003834-1 2.5191 2.8458 -0.3267 2.121 10.262 0 0 0 U004030 NM_026582.3 NM_026582.3 MGI:1915401 Gpr177 G protein-coupled receptor 177

Z00004618-1 3.0087 3.33479 -0.32609 2.118 15.204 0 0 0 U006614 XM_284175.6 MGI:95557 Flnc "filamin C, gamma (actin binding protein 280)"

Z00005050-1 2.7392 3.0649 -0.3257 2.116 11.373 0 0 0 U001184 NM_178653.2 NM_178653.2 MGI:1924486 Sccpdh saccharopine dehydrogenase (putative)

Z00010781-1 2.565 2.88919 -0.32419 2.109 10.766 0 0 0 U031500 NM_019958.2 NM_019958.2 MGI:1927469 Rgs17 regulator of G-protein signaling 17

Z00010400-1 2.541 2.8632 -0.3222 2.099 7.484 0 0 0 U139891 "H3095D09-3 NIA Mouse 15K cDNA Clone Set Mus musculus cDNA

clone H3095D09 3', mRNA sequence"

Z00007762-1 2.4647 2.78489 -0.32019 2.09 9.834 0 0 0 U022200 NM_007498.2 NM_007498.2 MGI:109384 Atf3 activating transcription factor 3

Z00005131-1 2.8939 3.21269 -0.31879 2.083 7.686 0 0 0 U059209 XM_972850.1 Igk-V19-17 similar to Ig kappa chain V-V region MPC11 precursor

Z00005116-1 3.3965 3.7151 -0.3186 2.082 19.039 0 0 0 U038335 NM_010415.1 NM_010415.1 MGI:96070 Hbegf heparin-binding EGF-like growth factor

Z00015931-1 2.77 3.08749 -0.31749 2.077 12.296 0 0 0 U028764 NM_011046.1 NM_011046.1 MGI:97513 Furin furin (paired basic amino acid cleaving enzyme)

Z00007505-1 2.98 3.2951 -0.3151 2.065 8.784 0 0 0 U036013 NM_008728.2 NM_008728.2 MGI:97373 Npr3 natriuretic peptide receptor 3

Z00016022-1 3.1023 3.4165 -0.3142 2.061 16.261 0 0 0 U028548 AF011750.1 MGI:1098729 Emp3 epithelial membrane protein 3

Z00006558-1 3.066 3.3797 -0.3137 2.059 16.092 0 0 0 U015989 NM_134082.3 NM_134082.3 MGI:2446173 Farp1 "FERM, RhoGEF (Arhgef) and pleckstrin domain protein 1

(chondrocyte-derived)"

Z00020415-1 2.6603 2.9737 -0.3134 2.057 8.456 0 0 0 U036777 NM_007929.2 NM_007929.2 MGI:1098726 Emp2 epithelial membrane protein 2

Z00012027-1 2.721 3.03429 -0.31329 2.057 9.715 0 0 0 U021297 NM_016917.1 NM_016917.1 MGI:1315204 Slc40a1 "solute carrier family 40 (iron-regulated transporter), member 1"

Z00019338-1 2.9655 3.2787 -0.3132 2.056 13.395 0 0 0 U000588 NM_007722.2 NM_007722.2 MGI:109562 Cxcr7 chemokine (C-X-C motif) receptor 7

Z00001201-1 3.5846 3.89769 -0.31309 2.056 17.879 0 0 0 U012897 NM_029564.1 NM_029564.1 MGI:1923531 Tax1bp3 Tax1 (human T-cell leukemia virus type I) binding protein 3

Z00003481-1 2.4909 2.8031 -0.3122 2.052 9.219 0 0 0 U003924 NM_018734.2 NM_018734.2 MGI:1926263 Gbp3 guanylate nucleotide binding protein 3

Z00017720-1 2.8602 3.1722 -0.312 2.051 10.765 0 0 0 U002498 NM_011170.1 NM_011170.1 MGI:97769 Prnp prion protein

Z00018966-1 2.5899 2.90129 -0.31139 2.048 7.935 0 0 0 U051828 NM_175446.3 NM_175446.3 MGI:2442284 B930008K04Rik RIKEN cDNA B930008K04 gene

Z00016300-1 2.6844 2.99399 -0.30959 2.039 6.483 0 0 0 U039691 XM_975492.1 MGI:2685563 Pcdh19 protocadherin 19

Z00012368-1 2.5678 2.8734 -0.3056 2.021 7.176 0 0 0 U016143 NM_008304.2 NM_008304.2 MGI:1349165 Sdc2 syndecan 2

Z00014876-1 3.2094 3.5139 -0.3045 2.016 9.726 0 0 0 U010601 NM_138304.2 NM_138304.2 MGI:1922850 Calml4 calmodulin-like 4
